# P-2132. A population-based assessment of infectious complications in patients with chronic lymphocytic leukemia (CLL) exposed to ibrutinib

**DOI:** 10.1093/ofid/ofaf695.2296

**Published:** 2026-01-11

**Authors:** Isabel H Gonzalez-Bocco, Seohyeon Im, Matthew Davids, Xiangmei Gu, Sophia Koo

**Affiliations:** Brigham and Women's Hospital, Boston, Massachusetts; Mass General Brigham-Salem Hospital, Salem, Massachusetts; Dana-Farber Cancer Institute, Boston, Massachusetts; Brigham and Women's Hospital, Boston, Massachusetts; Brigham and Women's Hospital, Dana-Farber Cancer Institute, Boston, MA

## Abstract

**Background:**

Ibrutinib, a Bruton tyrosine kinase inhibitor, is approved for treatment of frontline and relapsed/refractory chronic lymphocytic leukemia (CLL). While effective in the treatment of CLL, infectious complications are common, in many cases leading to treatment discontinuation.^7^ We performed a comprehensive, population-based assessment of infectious risks associated with ibrutinib among older patients with CLL.

**Table 1.**
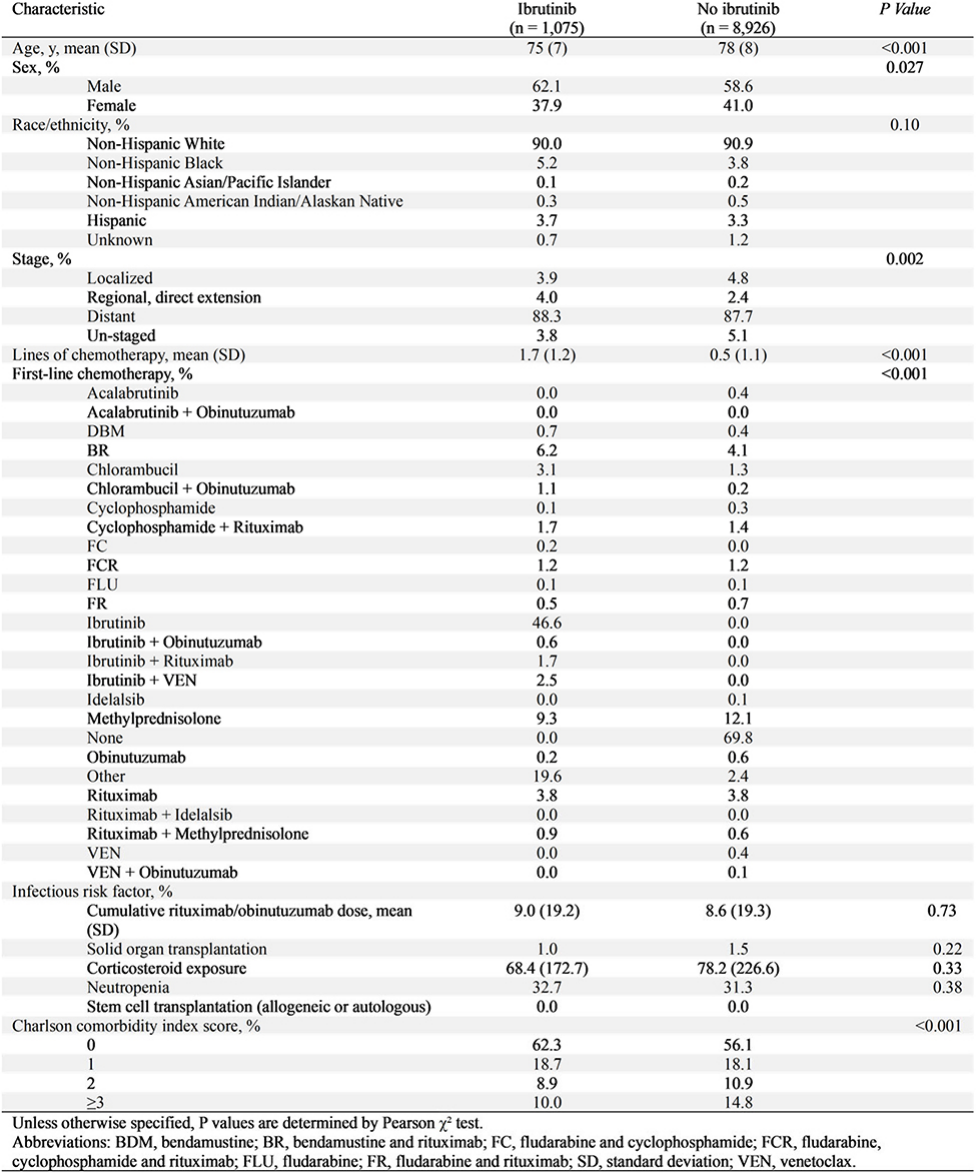
Cohort characteristics of patients with chronic lymphocytic leukemia (CLL) exposed vs. not exposed to ibrutinib.

**Table 2.**
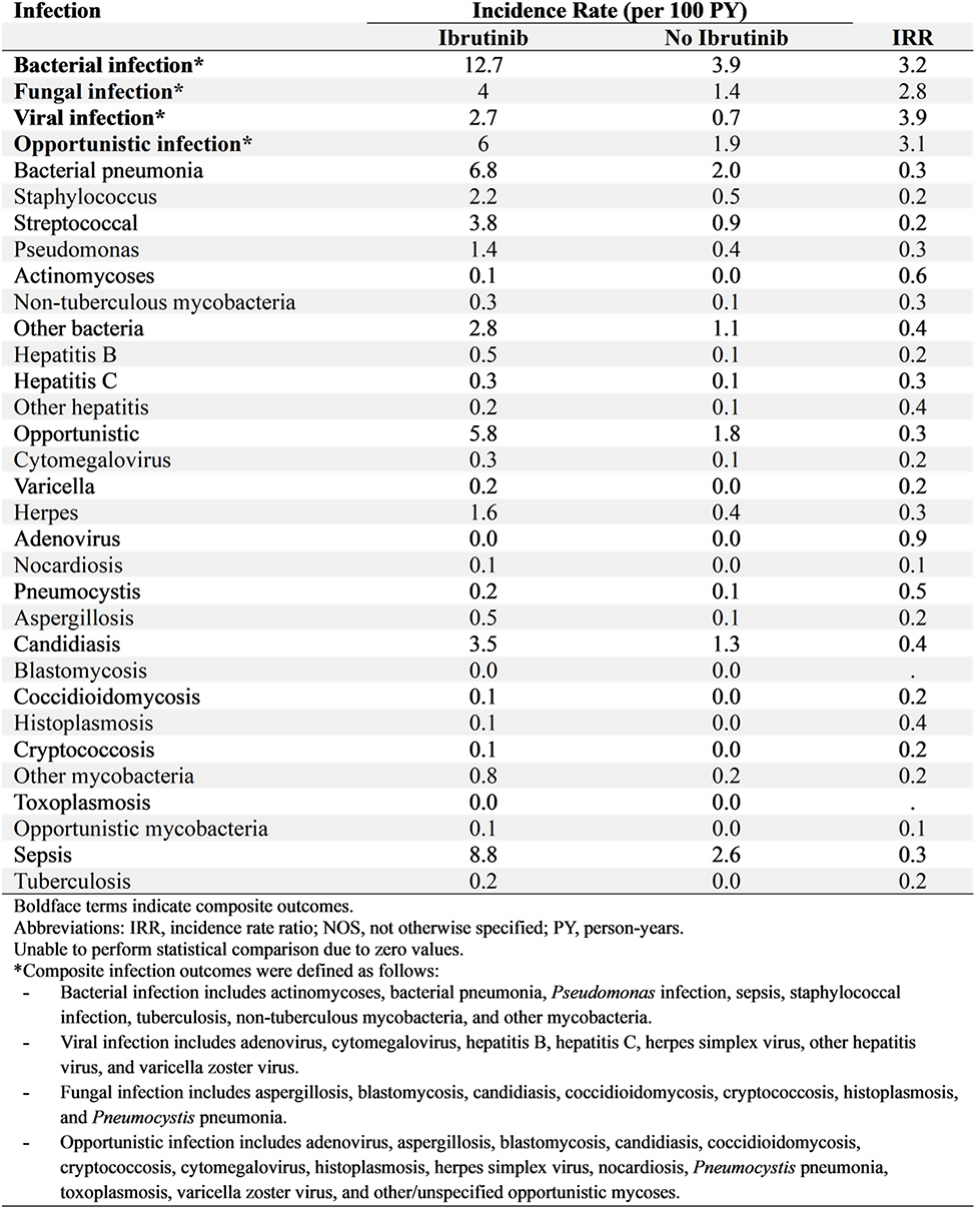
Crude incidence rates and incidence rate ratios of infectious complications among patients with CLL exposed vs. not exposed to ibrutinib.

**Methods:**

In this cohort study using the population-based Surveillance, Epidemiology, and End Results (SEER)-Medicare database, which encompasses 28% of Medicare beneficiaries with cancer in the United States, we identified 10,001 patients with CLL between 2009 and 2019 (Table 1). Eleven percent received ibrutinib-containing regimens. We compared patient characteristics and infection incidence rates between exposed and unexposed groups (Table 2). We assessed the infectious risks associated with ibrutinib using multivariate Cox proportional hazards models, adjusting for demographics, treatment characteristics, risk factors for infection, and receipt of antimicrobial prophylaxis.

**Table 3.**
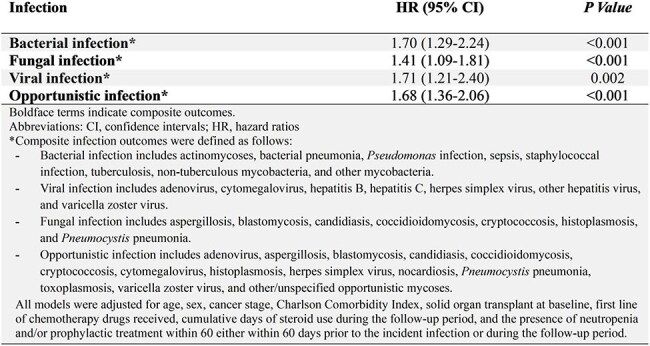
Adjusted hazard ratios for infection types in patients with CLL in those exposed vs. not exposed to ibrutinib.

**Results:**

After adjusting for potential confounders, ibrutinib was associated with a higher risk of viral (hazard ratio [HR] 1.71 [95% CI 1.21, 2.40]), bacterial (HR 1.70 [95% CI 1.29, 2.24]), fungal (HR 1.41 [95% CI 1.09, 1.81]), and opportunistic infections (HR 1.68 [95% CI 1.36, 2.06]) (Table 3). Higher Charlson comorbidity scores were independently associated with an increased risk of opportunistic infections (HR 1.25 [95% CI 1.14, 1.37, p < 0.001]).

**Conclusion:**

In this large, population-based study of patients with CLL, ibrutinib exposure was associated with a significantly increased risk of common bacterial, viral, and fungal infections, and opportunistic infections, although the incidence rates of these infections were generally not high enough to support the widespread use of antimicrobial prophylaxis in patients receiving ibrutinib. Targeted prophylaxis may be warranted in patients with a higher baseline risk of opportunistic infections due to additional comorbidities and concurrent exposure to other immunosuppressive agents.

**Disclosures:**

Matthew Davids, MD, AbbVie: Advisor/Consultant|AbbVie (Inst): Grant/Research Support|Adaptive Biotechnologies: Advisor/Consultant|Aptitude Health: Honoraria|Ascentage Pharma: Advisor/Consultant|Ascentage Pharma (Inst): Grant/Research Support|AstraZeneca: Advisor/Consultant|AstraZeneca (Inst): Grant/Research Support|Axis Medical Education: Honoraria|BeiGene: Advisor/Consultant|Bio Ascend: Honoraria|Bristol Myers Squibb: Advisor/Consultant|Curio Science: Honoraria|Galapagos NV: Advisor/Consultant|Genentech: Advisor/Consultant|Genentech (Inst): Grant/Research Support|Genmab: Advisor/Consultant|Janssen: Advisor/Consultant|Lilly: Advisor/Consultant|Medscape: Honoraria|MEI Pharma: Advisor/Consultant|Merck: Advisor/Consultant|MingSight: Advisor/Consultant|Novartis (Inst): Grant/Research Support|Nuvalent Inc: Advisor/Consultant|Peerview: Honoraria|Physicians’ Education Resource: Honoraria|PlatformQ Health: Honoraria|Plexus: Honoraria|Research to practice: Honoraria|Secura Bio: Advisor/Consultant|Secura Bio (Inst): Grant/Research Support|Takeda: Advisor/Consultant|TG Therapeutics: Advisor/Consultant|TG Therapeutics (Inst): Grant/Research Support|UptoDate: Royalties Sophia Koo, MD, SM, Ansun BioPharma: Grant/Research Support|Generate Biomedicines: Advisor/Consultant|GlaxoSmithKline: Grant/Research Support|Locus Biosciences: Grant/Research Support|Merck Sharp & Dohme: Grant/Research Support|Scynexis, Inc: Grant/Research Support|Vertex Pharmaceuticals: Advisor/Consultant

